# Fungal keratitis in Lattice dystrophy

**DOI:** 10.4103/0301-4738.60097

**Published:** 2010

**Authors:** Samrat Chatterjee, Deepshikha Agrawal

**Affiliations:** Cornea and Anterior Segment Services, MGM Eye Institute, 5^th^ Mile, Vidhan Sabha Road, Raipur, India

**Keywords:** *Alternaria alternata*, corneal dystrophy, fungal keratitis, lattice dystrophy, natamycin

## Abstract

We report a case of fungal keratitis occurring in a patient with lattice dystrophy. A 57-year-old farmer presented with a corneal ulcer following probable entry of paddy husk in the right eye, of one month duration. Corneal scraping revealed pigmented fungal filaments while culture grew *Alternaria alternata*. Treatment with 5% natamycin eye drops and 1% atropine healed the infection in four weeks. We would like to draw attention to the fact that the cornea in lattice dystrophy is prone to frequent erosions and is a compromised epithelial barrier to invasion by microorganisms. Patients must be made aware of this fact and should seek attention at the earliest following any trivial trauma. Management of minor corneal abrasions in them should be directed at healing the epithelium with adequate lubricants and preventing infection with topical antibiotic prophylaxis.

Lattice dystrophy of the cornea is a bilateral, inherited, primary, localized corneal amyloidosis characterized by subepithelial opacities, stromal white dots, refractile filamentary lines and stromal haze giving rise to recurrent corneal erosions and irregularity of the epithelium with accompanying decrease in visual acuity.[1] Recurrent erosion and an unhealthy epithelium in lattice dystrophy may predispose the cornea to microbial infections.[2] Few such cases have been reported in the literature with bacterial and viral infections.[2–4] We report an uncommon case of fungal keratitis in a patient with lattice dystrophy.

## Case Report

**Figure 1:**
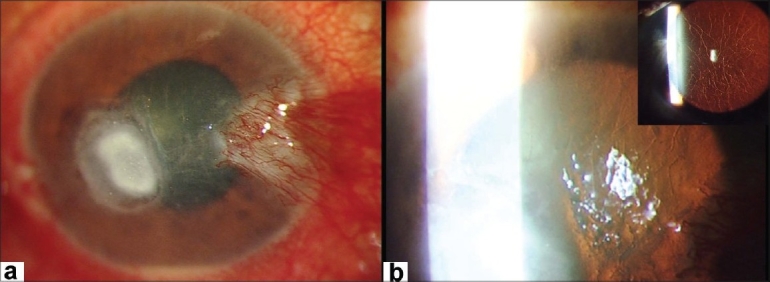
Central corneal ulcer and nasal pterygium in the right eye; (a) with refractile lines of lattice dystrophy extending to periphery seen in retroillumination; (b) Inset shows nasal pterygium and lattice lines in the left eye

**Figure 2:**
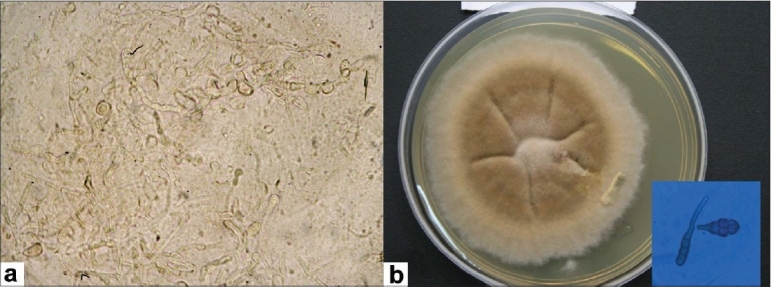
Ten per cent potassium hydroxide preparation showing brown pigmented fungal filaments; (a), brown wooly colony in Sabouraud's dextrose agar medium; (b) with ovoid, short, beak-like multicellular conidia of *Alternaria alternata* (inset)

**Figure 3:**
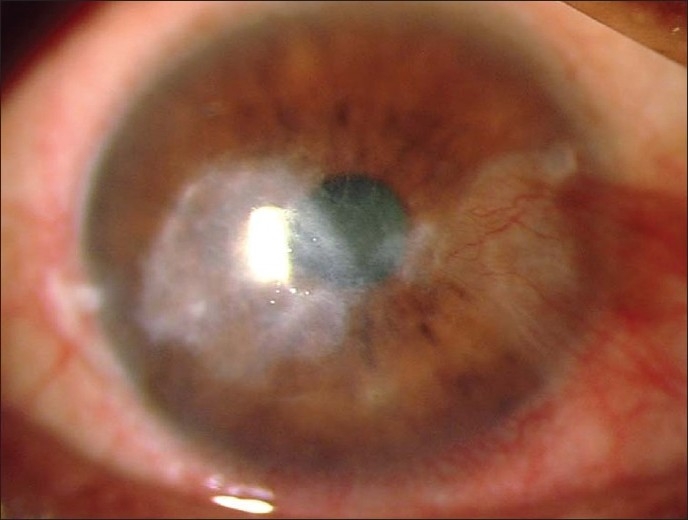
After healing, the cornea in the right eye shows a corneal scar with intact epithelial surface

A 57-year-old farmer presented with complaints of pain, redness, watering and reduced vision in the right eye, of one-month duration. There was a vague history of entry of paddy husk in the eye. He also gave a history of repeated episodes of foreign body sensation, pain and watering in both the eyes since five years. His visual acuity in the right eye was counting fingers at 2 m while in the left eye was 20/20. There was nasal pterygium in both eyes. There were no signs of blepharitis or meibomitis. There was a para-central epithelial defect with dry, white, full-thickness stromal infiltrate of 3 × 2.5 mm diameter [Fig. 1a]. There were multiple linear refractile lines consistent with lattice-lines in the corneal stroma extending to the periphery [Fig. 1b]. There was hypopyon, the pupil was mid-dilated and the lens showed nuclear sclerosis. Slit-lamp examination of the left eye [Fig. 1b, inset] also revealed linear refractile lines in the corneal stroma extending to the periphery and nuclear sclerosis. Intraocular pressure was digitally normal in the right eye and was 12 mmHg in the left eye. Fundus examination was unremarkable in both eyes. Corneal scraping revealed multiple septate brown fungal filaments in 10% potassium hydroxide preparation [Fig. 2a]. No bacteria were seen on Gram's stain. He was diagnosed with fungal keratitis in the right eye and lattice dystrophy with pterygium in both eyes. Epithelial debridement was done with No. 15 blade, and natamycin 5% eye drop every half hour, atropine 1% eye drop three times and oral Ketoconazole 200 mg twice daily (after a normal liver function test) was advised. Culture grew brown wooly confluent colony in Sabouraud's dextrose agar and spores stained with lactophenol cotton blue revealed them to be of *Alternaria alternata* [Fig. 2b]. The ulcer healed after four weeks leaving behind a moderately dense stromal scar [Fig 3]. Four months later his visual acuity was 20/100.

## Discussion

Recurrent erosions in lattice dystrophy have been postulated to be due to abnormal basement membrane complexes that provide poor epithelial stromal adhesions or an abnormal basal epithelial cell wall incapable of providing normal structural integrity to the epithelial layer.[2] Thus the corneas in lattice dystrophy with recurrent erosions are susceptible to infections due to breach in the normal epithelial barrier. Further, because of compromised epithelial layer, healing is delayed. Goodall *et al*.,[2] have reported two patients with mixed bacterial infections while others have reported patients with bacterial and viral infection or of unknown etiology.[3–5] Thus occasionally the chronic course of lattice dystrophy may be altered to a sight-threatening ocular emergency with infection by pathogenic microorganisms.

Our patient suffered from an infection with *Alternaria alternata* following probable entry of paddy husk. Although ubiquitous in the environment, *Alternaria* species are not a common ocular pathogen in the etiology of fungal keratitis in India.[6] Minor trauma to the eye due to paddy is a common risk factor for keratitis in agricultural communities, in whom the incidence of fungal keratitis is highest in India.[6] However, all such incidents do not result in ulcer unless there is a breach in the corneal epithelium, delay in healing that provides the “window of opportunity”,[7] lodgment of the microorganism and development of subsequent ulceration. Often it may be difficult to ascertain whether the sequel of corneal ulceration in this group of patients is because of direct trauma or because of the poorly healing epithelium. Clinically, it may also be difficult in the early stages to differentiate corneal erosions from infections. Further symptoms of recurrent corneal erosion in lattice dystrophy need to be distinguished from symptoms of lid margin disease which is commonly present in the general population. Nevertheless the compromised epithelium in lattice dystrophy is prone to frequent erosions and poor healing and thus provides the necessary *milieu* for the development of fungal keratitis.

In conclusion we would like to draw the attention of all ophthalmologists to the risk of microbial keratitis in patients with lattice and other epithelial and stromal dystrophies which compromises the corneal epithelial barrier. Patients should be advised about using protective glasses during their work to avoid entry of foreign body or mild trauma. Further, those suffering from mild ocular surface abrasion following trauma may benefit from prophylaxis with topical antibiotics[7] and sufficient lubricants to promote healing.
